# Performance of the CareStart™ G6PD Deficiency Screening Test, a Point-of-Care Diagnostic for Primaquine Therapy Screening

**DOI:** 10.1371/journal.pone.0028357

**Published:** 2011-12-02

**Authors:** Saorin Kim, Chea Nguon, Bertrand Guillard, Socheat Duong, Sophy Chy, Sarorn Sum, Sina Nhem, Christiane Bouchier, Magali Tichit, Eva Christophel, Walter R. J. Taylor, John Kevin Baird, Didier Menard

**Affiliations:** 1 Malaria Molecular Epidemiology Unit, Pasteur Institute of Cambodia, Phnom Penh, Cambodia; 2 National Center for Parasitology, Entomology, and Malaria Control, Phnom Penh, Cambodia; 3 Medical Laboratory, Pasteur Institute of Cambodia, Phnom Penh, Cambodia; 4 Genomic Platform, Institut Pasteur, Paris, France; 5 WHO Regional Office for the Western Pacific, Manilla, Philippines; 6 Service de Médecine Internationale et Humanitaire, Hopitaux Universitaires de Genève, Geneva, Switzerland; 7 Eijkman Oxford Clinical Research Unit, Jakarta, Indonesia; 8 Centre for Tropical Medicine, Nuffield Department of Clinical Medicine, University of Oxford, Oxford, United Kingdom; Laboratory of Malaria Immunology and Vaccinology, United States of America

## Abstract

Development of reliable, easy-to-use, rapid diagnostic tests (RDTs) to detect glucose-6-phosphate dehydrogenase (G6PD) deficiency at point of care is essential to deploying primaquine therapies as part of malaria elimination strategies. We assessed a kit under research and development called CareStart™ G6PD deficiency screening test (Access Bio, New Jersey, USA) by comparing its performance to quantitative G6PD enzyme activity using a standardized spectrophotometric method (‘gold standard’). Blood samples (n = 903) were collected from Cambodian adults living in Pailin province, western Cambodia. G6PD enzyme activities ranged from 0 to 20.5 U/g Hb (median 12.0 U/g Hg). Based on a normal haemoglobin concentration and wild-type G6PD gene, the normal values of G6PD enzymatic activity for this population was 3.6 to 20.5 U/g Hg (95^th^ percentiles from 5.5 to 17.2 U/g Hg). Ninety-seven subjects (10.7%) had <3.6 U/g Hg and were classified as G6PD deficient. Prevalence of deficiency was 15.0% (64/425) among men and 6.9% (33/478) among women. Genotype was analyzed in 66 G6PD-deficient subjects and 63 of these exhibited findings consistent with Viangchang genotype. The sensitivity and specificity of the CareStart™ G6PD deficiency screening test was 0.68 and 1.0, respectively. Its detection threshold was <2.7 U/g Hg, well within the range of moderate and severe enzyme deficiencies. Thirteen subjects (1.4%, 12 males and 1 female) with G6PD enzyme activities <2 U/g Hg were falsely classified as “normal” by RDT. This experimental RDT test here evaluated outside of the laboratory for the first time shows real promise, but safe application of it will require lower rates of falsely “normal” results.

## Introduction

In the context of malaria elimination, vector control measures e.g. long lasting bednets and indoor residual spraying along with prompt diagnosis and treatment of malaria infected patients are the most effective tools currently available [1]. Antimalarial drugs are seen as crucial to eliminate malaria and the focus is on the role of drugs to block malaria transmission by killing gametocytes and reducing the pool of liver hypnozoites of *Plasmodium vivax* and *P. ovale* [2]. 8-aminoquinolines like primaquine (and tafenoquine, a drug in phase III clinical trials) remain the only transmission-blocking drugs available. While Artemisinin Combined Therapies (ACTs) reduce gametocytogenesis in *P. falciparum* malaria by killing young gametocytes, they lack activity against mature gametocytes [3]. Therefore, primaquine remains a vitally important tool to block the transmission of parasites, especially in areas like Cambodia, where artemisinin resistant parasites are well documented [4], [5], [6]. Moreover, the 8-aminoquinolines are the only effective drugs capable of killing *P. vivax* and *P. ovale* liver hypnozoites, thereby, preventing relapses [7]. However, primaquine and tafenoquine cause predictable, intravascular haemolysis in individuals with glucose-6-phosphate dehydrogenase (G6PD) deficiency. Primaquine, at the transmission blocking dose (single dose, 0.75 mg/kg), has been shown to cause haemolysis in *P. falciparum*-infected African children without G6PD deficiency [8] and to cause a greater initial fall in haemoglobin following treatment compared to ACT alone [3]. High dose primaquine also caused haemolysis in healthy, G6PD normal individuals [9]. This dose of primaquine causes only slight drops in haemoglobin in healthy African-American volunteers having the A- variant, but in healthy volunteers having the B- Mediterranean variant, a hemolysis of 25% of red blood cells occurs [10]. Thus, risk of hemolysis with primaquine varies with status of both infection and G6PD deficiency variant.

The conversion of NADP+ to NADPH by G6PD is the rate-limiting reaction in the pentose phosphate pathway, the primary source of reducing potential for the glutathione redox flux which serves to as the primary protection of erythrocytes against oxidative stress. Numerous drugs and chemicals such as primaquine, foods (fava beans) or stress (infections) can induce hemolytic anemia in G6PD-deficient individuals. G6PD deficiency is the second most common hereditary enzyme deficiency which affects approximately 400 million people worldwide [11] and is distributed in areas of current and previous endemic malaria [12]. This human enzyme defect is caused by mutations in the G6PD gene located on chromosome Xq28; thus, transmission of the genetic defect is X linked. Hemizygote males are most affected and homozygous females least affected; both are prone to red cell haemolysis. Heterozygote females have mixed G6PD normal and deficient red cells and their susceptibility to haemolysis depends on the balance between the expression of the normal and abnormal X chromosomes [13]. The highest frequencies are detected in Africa, Southeast Asia, Central and Southern Pacific islands, the Mediterranean region, and in the Middle East [14]. There are some 140 different genotypes of G6PD deficiency [15] with corresponding enzyme activity phenotypes that vary from mild to severe. Primaquine sensitivity phenotypes are known only in 3 variants; African A-, Mahidol, and Mediterranean B-, representing mild, moderate and severe sensitivity, respectively. Correlation between severity of enzyme activity loss and sensitivity to primaquine is believed to exist but has not been confirmed with clinical observations.

In Cambodia, G6PD deficiency is common, with a prevalence ranging from 13.4% to 26.1% in males and 3.1% to 4.3% in females, depending on the sampled population [16], [17]. G6PD-Viangchan is the most frequent variant [16], [18].

G6PD deficiency may be diagnosed by a variety of spectrophotometric enzyme activity assays or DNA-based detection of specific mutations. These tests, however, require relatively sophisticated laboratory capacities. Qualitative tests for this disorder based upon reduction of NADP+ by G6PD have been used for screening patients in the absence of laboratory facilities prior to administration of primaquine therapy. Since 1979, the fluorescent spot test is recommended as the most suitable method for screening in the field, despite the need for an UV lamp, water bath incubator, and micropipettor [19], [20]. Such equipment and the skills to use them are rarely available in malaria endemics areas [8]. Consequently, providers in such settings invite substantial risk of harm by prescribing primaquine therapy and the drug is thus rarely used. Rapid diagnostic tests (RDTs) are an alternative and attractive option because they are generally easy to use and could be used alongside malaria RDTs. However, the only available test named BinaxNOW G6PD test™ (ref 780-000, Binax, Inc., Maine, USA) which has been recently evaluated, presents a major drawback with the need to perform the test within a temperature ranging from 18 to 25°C, imposing limited usefulness in tropical malaria-endemic countries [21]. AccessBio (New Jersey, USA) has developed a new experimental RDT called CareStart™ G6PD deficiency screening test and its first assessment outside of their laboratories is reported here, by comparing its performance to the gold standard, quantitative spectrophotometric estimation of G6PD enzyme activity [14].

## Materials and Methods

### Ethics statement

The study protocol was reviewed and approved by the National Ethics Committee for Health Research of the Ministry of Health of Cambodia (approval number 028 NECHR). Informed written consent was provided by all individuals before inclusion in the study and all investigations were conducted according to the principles expressed in the Declaration of Helsinki. Results for each patient (according to the quantitative G6PD activity test) were given to the local Ministry of Health staff of Pailin district involved in the study. Participants were been notified of their G6PD status. For deficient people, information regarding their deficit was provided.

### Population and Sample collection

**Figure 1:**
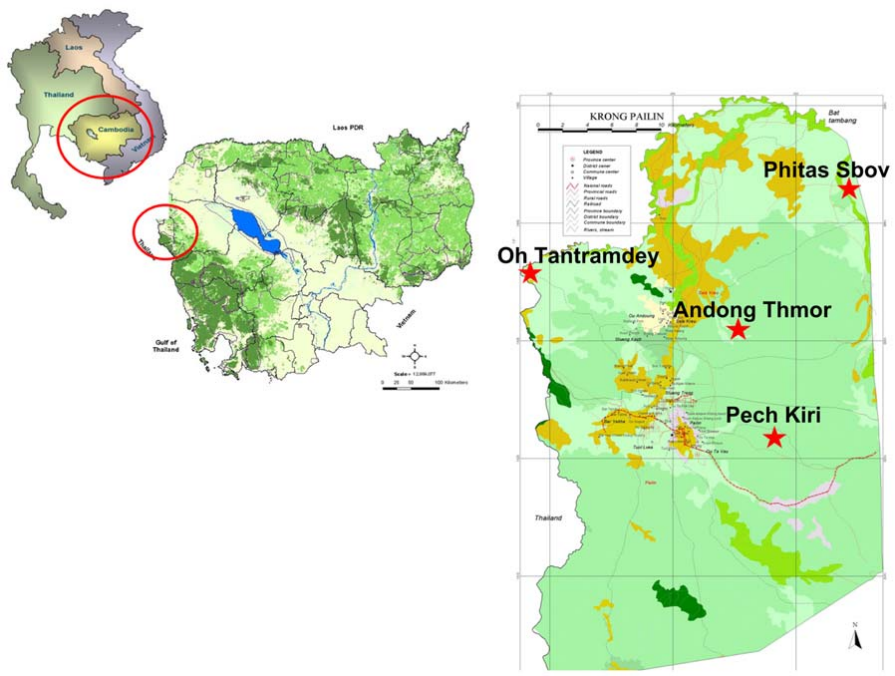
Map of Cambodia locating the four selected villages in Pailin province, Cambodia, 2010.

A cross sectional survey was conducted in four malaria endemic villages in Pailin Province in western Cambodia (Figure 1). After informed consent was obtained, 2 ml of venous blood was collected in EDTA-tube from healthy participants over 18 years of age. Individuals with illness that affected competency to give informed consent, along with pregnant or lactating women were excluded from the study. Demographic information was gathered by questionnaire. After collection, the fresh blood was used within 15 minutes to perform the CareStart™ G6PD deficiency screening test in the field, according to manufacturers' instructions, and then sent to the Malaria Molecular Epidemiology Unit at Pasteur Institute in Cambodia (IPC) in 4°C cool box within 24 hours. At IPC, the quantitative determination of G6PD enzymatic activity was performed using 100 µl of fresh blood. The remaining blood samples were centrifuged and aliquots of plasma, buffy coat and red blood cells pellets were stored at −20°C for DNA analysis.

### CareStart™ G6PD deficiency screening test

**Figure 2:**
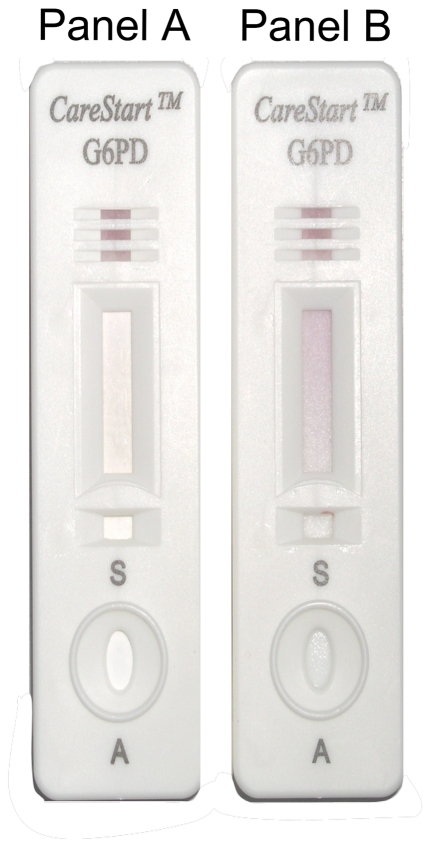
Design of the CareStart™ G6PD deficiency screening test and interpretation of the results. Panel A, no color change for sample with deficient G6PD enzymatic activity; Panel B, distinct purple color for sample with normal G6PD enzymatic activity.

The CareStart™ G6PD deficiency screening test was provided by the manufacturer (AccessBio, New Jersey, USA). The kit contains the test strip encased in a flat plastic cassette (containing a buffer well, a sample well and a result window), a sample pipette, the assay buffer, an alcohol pad and a blood lancet. This RDT-format test, which is not yet commercially available, is a qualitative enzyme chromatographic test, based on the reduction of colorless nitro blue tetrazolium dye to dark colored formazan. Following the manufacturer instructions, two microliters of blood were added into the sample well and two drops of buffer into the buffer well. Test results were read visually after 10 minutes. Samples with normal G6PD activity produce a distinct purple color background in the result window while no color change was observed at the test read time for samples with deficient G6PD activity (Figure 2). Samples with a pale purple color background were classified as normal. Long term temperature stability of the CareStart™ G6PD deficiency screening test for providing information about RDT survival when used and stored outside the specified temperatures was assessed following a modified procedure described for malaria RDT lot testing Quality Control [22] by using quality control (QC) materials (normal level, ref. G6888 deficient level ref. G5888 from Trinity Biotech). Briefly, RDT-format tests were stored in incubators (35°C, 45°C and 55°C) with daily temperature control. QC were repeated at D5, D10, D20, D30, D60 and D90 for RDT-format tests stored at 35°c and 45°C and at H4, H12, H24, H48 and H72 for RDT-format tests stored at 55°C.

### Quantitative determination of G6PD activity

Quantitative determination of G6PD activity was performed on the fresh blood within a maximum of 48 h after the collection, using the Trinity Biotech quantitative G6PD assay™ (Ref. 345-UV, Trinity Biotech, St. Louis, USA) adapted on the Integra 400™ automate (Roche diagnostic, Meylan, France), according to manufacturer's instructions. Briefly, the procedure is a modification of the spectrophotometric methods of Kornberg and Horecker [23] and of Lohr and Waller [24], involving the reduction of NADP to NADPH by the G6PD enzyme in the presence of glucose-6-phosphate and maleimide, acting as an inhibitor of erythrocyte 6-phosphogluconate dehydrogenase (6-PGDH). The formation of NADPH is proportional to the G6PD activity and is measured spectrophotometrically as an increase in absorbance at 340 nm. Reliability of test results were monitored by calibration and the use of three different controls provided by Trinity Biotech (deficient level ref. G5888, intermediate level ref. G5029 and normal level, ref. G6888) within each run. Runs were considered valid if control values fell within the given range. G6PD activities were expressed in terms of amount of hemoglobin (U/g Hg). Determination of hemoglobin content of the EDTA sample was performed on a CellDyn 3200™ analyser (Abbott, Rungis, France) after standardization with three different controls. The investigator and laboratory technicians running the quantitative enzymatic assay were masked to the results of the CareStart™ G6PD deficiency screening test.

### Detection of G6PD variants

Following the quantitative determination of G6PD activity and using the normal ranges provided by Trinity Biotech (www.trinitybiotech.com/Product%20Documents/345-29%20EN.pdf), sequencing of the G6PD gene was performed on all samples classified as deficient (<4.6 U/g Hg) and a randomly selected number of samples classified as normal (≥4.6 U/g Hg).

### DNA extraction

Human DNA was extracted from the buffy coat using the QIAamp DNA Blood Mini Kit™ (Qiagen, Courtaboeuf, France), according to the manufacturer's instructions.

### DNA fragment amplification

Primers were designed to PCR amplify exons of the G6PD gene, as shown in Table 1. The complete exonic regions were generated in 8 fragments ranging between 221 and 848 bp. PCR was performed in a 55 µL reaction containing 0.4 µM of each specific primer, 0.25 mM of each dNTP, 1.5 to 2.5 mM MgCl_2_, and 1.5 unit of FirePol Taq polymerase™ (Solis Biodyne, Tartu, Estonia). PCR cycling conditions were as follows: heating at 94°C for 5 min, followed by 40 cycles of heating at 94°C for 30 s, 56–58°C for 30–90 s and 72°C for 60–150 s, and a final extension period at 72°C for 10 min.

**Table 1:** Primers sequence, annealing temperatures and size of PCR products used to amplify and sequence exons of the G6PD gene.

Exons	Primer name	Sequence (5′-3′)	Hybridization T°	Size of PCR products
Exon 2	PCR_G6PD_Ex2_F	TGAAGGCTGCCTAGGAGAGA	58°C	494 bp
	PCR_G6PD_Ex2_R	CAGGTAGAGCCGGGATGAT		
Exons 3–4	PCR_G6PD_Ex3–4_F	TGTCCCCAGCCACTTCTAA	58°C	400 bp
	PCR_G6PD_Ex3–4_R	GGAGAGGAGGAGAGCATCC		
Exon 5	PCR_G6PD_Ex 5_F	CGGGGACACTGACTTCTG	56°C	500 bp
	PCR_G6PD_Ex 5_R	ACGCTGCCACCTTGTGGT		
Exons 6–7	PCR_G6PD_Ex 6–7_F	ACACAAGGCACGGGAGGT	58°C	697 bp
	PCR_G6PD_Ex 6–7_R	GAGGAGCTCCCCCAAGATAG		
Exon 8	PCR_G6PD_Ex 8_F	CCCTTGAACCAGGTGAACAG	58°C	221 bp
	PCR_G6PD_Ex 8_R	TCAGTGCCTCGTCACAGATG		
Exon 9	PCR_G6PD_Ex 9_F	CCTGAGGGCTGCACATCT	58°C	363 bp
	PCR_G6PD_Ex 9_R	GTGCGTGAGTGTCTCAGTGG		
Exons 10–11–12	PCR_G6PD_Ex 10–12_F	TGAGACACTCACGCACTGGT	58°C	752 bp
	PCR_G6PD_Ex 10–12_R	TGAGGTAGCTCCACCCTCAC		
Exon 13	PCR_G6PD_Ex 13_F	TTATGGCAGGTGAGGAAAGG	58°C	848 bp
	PCR_G6PD_Ex 13_R	GAAGTGGGTCCTCAGGGAAG		

### Sequencing reactions

After purification by filtration with a NucleoFast 96 PCR plate™ (Macherey-Nagel, Düren, Germany), sequencing reactions were performed for both strands by using the ABI Prism BigDye Terminator cycle sequencing ready reaction kit™ run on a 3730 xl genetic analyzer™ (Applied Biosystems, Courtaboeuf, France). Electrophoregrams were visualized and analyzed with CEQ2000 genetic analysis system software™ (Beckman Coulter, Villepinte, France). Nucleotide sequences were compared to the sequence of G6PD in GenBank (accession No. X55448) to identify mutations.

### Statistical analysis

Data were entered and verified using Microsoft Excel™ software, and analyzed using EpiInfo 6.04™ software (CDC, Atlanta, USA) and MedCalc™ version 11.6.1 software (MedCalc, Mariakerke, Belgium). The Mann-Whitney U test or Kruskal-Wallis method were used for non-parametric comparisons, and Student's t test or one-way analysis of variance for parametric comparisons. For categorical variables, Chi-squared or Fisher's exact tests were used to assess significant differences in proportions. *P* values<0.05 indicated statistically significant differences.

The mean, median, standard deviation and ranges were determined for all G6PD enzyme activity values. The normal values of G6PD enzymatic activity in Cambodian adults were determined by gender from a subset of random samples classified as normal using the quantitative G6PD assay (≥4.6 U/g Hg) and meeting the following criteria: hemoglobin concentration >12 g/dL, and absence of genetic polymorphism compared to the reference sequence: *H. sapiens* G6PD gene for glucose-6-phosphate dehydrogenase, accession No. X55448. Distribution of normal G6PD activity was evaluated by gender using Kolmogorov-Smirnov test.

Subjects were also classed according to the WHO classification for G6PD, using the population derived mean G6PD enzyme activity as the reference for residual activity [14]: (i) Class I (very severely deficient (associated with chronic non-spherocytic hemolytic anaemia, <1% residual activity, <0.12 U/g Hg), (ii) Class II (severely deficient, 1 to 10% residual activity, 0.13–1.2 U/g Hg), (iii) Class III (moderately deficient, 10 to 60% residual activity, 1.3–7.1 U/g Hg), (iv) Class IV (normal activity, 60 to 150% residual activity, 7.2–17.7 U/g Hg) and (v) Class V (increased activity, >150% residual activity, >17.7 U/g Hg).

To assess the performance of the CareStart™ G6PD deficiency screening test, individuals were grouped as G6PD deficient or normal, using the normal values of G6PD enzymatic activity determined in our Cambodian population. Standard diagnostic test measures were determined: Sensitivity (Se) was the probability that the CareStart™ G6PD deficiency screening test classify individuals as deficient among individuals classified as deficient by using the quantitative G6PD assay (true positive rate). Specificity (Sp) was the probability that the CareStart™ G6PD deficiency screening test classify individuals as normal among individuals classified as normal by using the quantitative G6PD assay (true negative rate). Positive Likelihood Ratio (PLR) was considered as the ratio between the true positive rate and the false positive rate ( = sensitivity/[1−Specificity]) and Negative Likelihood Ratio (NLR) as the ratio between the false negative rate and the true negative rate ( = [1−Sensitivity]/Specificity). Positive Predictive Value (PPV) was the probability that G6PD deficiency was present in individuals when the CareStart™ G6PD deficiency screening test classified individuals as deficient and Negative Predictive Value (NPV), the probability that G6PD deficiency was not present in individuals when the CareStart™ G6PD deficiency screening test classified individuals as normal. PPV and NPV were calculated based on the prevalence (i.e. prior probability, PP) of G6PD deficiency found in our sampling population as following: PPV = Se×PP/Se×PP+(1−Sp)×(1−PP) and NPV = Sp×(1−PP)/Sp×(1−PP)+(1−Se)×PP.

## Results

### Study population and distribution of the G6PD enzymatic activity

From October to December 2010, nine hundred three blood samples were collected in 4 villages in Pailin Province (Phitas Sbov n = 80; Andong Thmor, n = 127; Oh Tantramdey, n = 93 and Pech Kiri, n = 603). No significant differences were observed between villages for sex ratio M/F (mean = 0.89, *P* = 0.74) and age (mean = 23.9 years, *P* = 0.53), except for hemoglobin level (mean = 12.9 g/dL) which was lower in Pech Kiri compared to Phitas Sbov (12.8 g/dL vs. 13.4 g/dL, *P* = 0.03).

**Figure 3:**
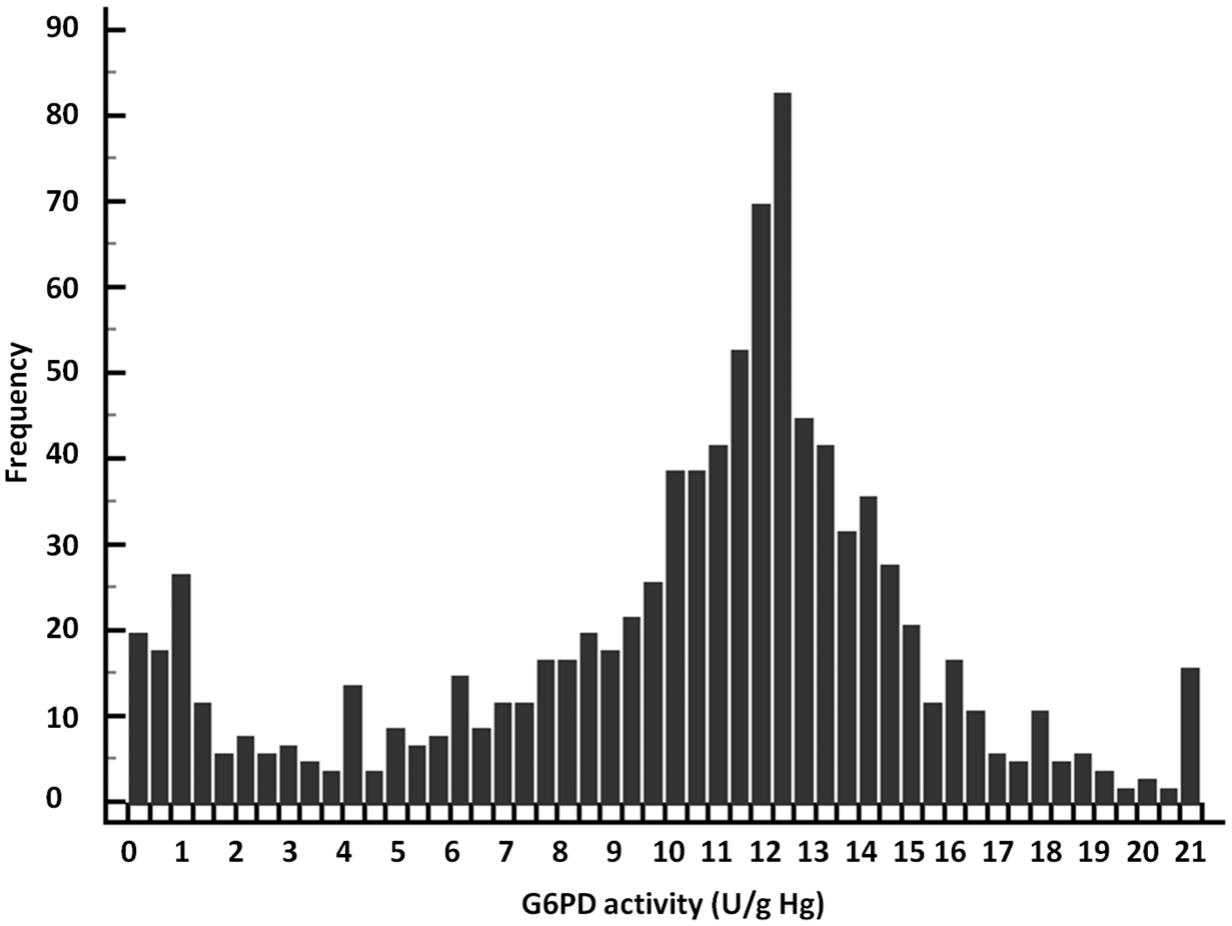
Distribution of the G6PD enzymatic activity (U/g Hb) values of 903 individuals enrolled in four villages of the Pailin province, Cambodia, 2010.

G6PD enzymatic activity values ranged from 0 to 20.9 U/g Hg with arithmetic mean of 10.9 U/g Hg (95%CI: 10.6–11.2), SD of 4.6 U/g Hg and median of 12.0 U/g Hg (95%CI: 11.9–12.2). The distribution of G6PD enzymatic activity (Figure 3) visually displays two different populations. No significant differences were observed between gender and age. However, significant differences were found between villages (*P*<0.01): the lowest mean was observed in Phitas Sbov (7.4 U/g Hg, SD = 3.9 U/g Hg) and the highest in Pech Kiri (11.7 U/g Hg, SD = 5.2 U/g Hg).

### Normal values of G6PD enzymatic activity in Cambodian adults

**Figure 4:**
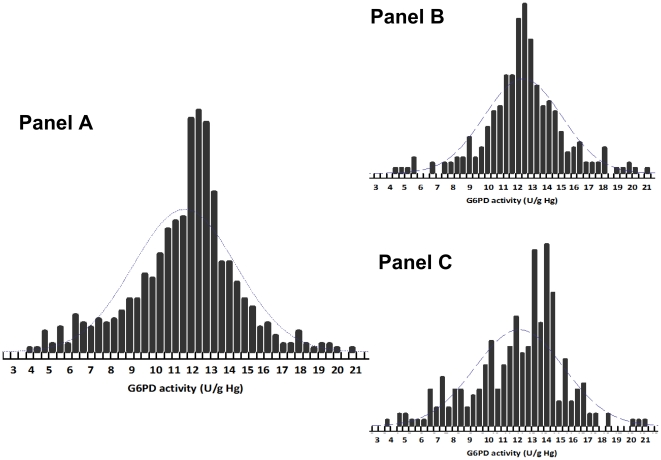
Distribution of the G6PD enzymatic activity (U/g Hb) reference values according to gender, of 147 G6PD-normal individuals (see criteria in Statistical analysis section) enrolled in four villages of the Pailin province, Cambodia, 2010. (Panel A: Total population, Panel B: Male population and Panel C: Female population).

Based on 147 normal samples (74 males and 73 females) as described in Statistical analysis section, the normal range of G6PD activity for all subjects was 3.6 to 20.5 U/g Hb; the lower limit of normal was slightly lower for females (Table 2). G6PD values were normally distributed for males (P = 0.05) (Figure 4, Panel B). Mean G6PD enzymatic activities were significantly higher for males (*P* = 0.005).

**Table 2:** Reference values for G6PD enzymatic activity (U/g Hg), Pailin province, Cambodia, 2010.

Reference values for G6PD enzymatic activity	Total	Male	Female
**N**	147	74	73
**Range**	3.6–20.5	4.3–20.5	3.6–18.9
**Mean (95%CI), U/g Hg**	11.8 (11.6–12.1)	12.5 (12.2–12.8)	11.3 (11.0–11.7)
**SD, U/g Hg**	2.7	2.5	2.6
**Median (95%CI), U/g Hg**	12.2 (12.0–12.4)	12.5 (12.3–12.7)	12.0 (11.5–12.0)
**95^th^ percentiles, U/g Hg**	5.5–17.2	6.5–17.9	5.6–15.5

N: Number of individuals; SD: Standard Deviation; 95%CI: 95% Confidence Interval.

### Prevalence & classification of G6PD deficiency

Based on the normal values of G6PD enzymatic activity defined in our Cambodian population, the prevalence of G6PD deficiency was 10.7% (97/903). Significant differences were observed between the sexes: 64/425 (15.0%, CI95%: 11.6%–19.2%) for males, and 33/478 (6.9%, CI95%: 4.7%–9.7%) for females, *P*<0.001) and villages (9.3% in Pech Kiri, 10.2% in Andong Thmor, 12.9% in Oh Tantramdey and 20.0% in Phitas Sbov, *P*<0.001 ). According to the WHO classification, 1.2% (11/903) individuals were classified as Class I, 5.6% (51/903) as Class II, 11.0% (99/9093) as Class III, 76.6% (691/903) as Class IV, and 5.6% (51/903) as Class V.

### Genotyping data and major variants

Four different variants were detected among the 64 samples used for sequencing of the G6PD gene. The most prevalent was G6PD-ViangChan (frequency 95.5%, 61/64, 871G>A, 1311C>T, IVS11 nt93T>C) followed by G6PD-Canton (frequency 1.5%, 1/64, 1376G>T), G6PD-Mahidol (frequency 1.5%, 1/64, 487G>A) and G6PD-Valladolid (frequency 1.5%, 1/64, 406C>T, 1311C>T). Means ± SD of G6PD enzymatic activity in hemizygous males were 1.1±1.9 U/g Hg for the G6PD-ViangChan variant (N = 38), 0.2 U/g Hg for the G6PD-Canton variant (N = 1) and 3.3 U/g Hg for the G6PD-Valladolid variant (N = 1). For heterozygous females, means ± SD of G6PD enzymatic activity were 4.4 U/g Hg for the G6PD-Mahidol variant (N = 1) and 2.9±1.9 U/g Hg for the G6PD-ViangChan variant (N = 10). For homozygous females, mean ± SD of G6PD enzymatic activity of the G6PD-ViangChan variant (N = 13) was 1.1±0.9 U/g Hg. Global distribution of the silent mutations, 1311C>T in exon 11 and T>C in nt93 in IVS11 in people with normal G6PD enzymatic activity was 42.8%.

### Performance of the CareStart™ G6PD deficiency screening test

**Figure 5:**
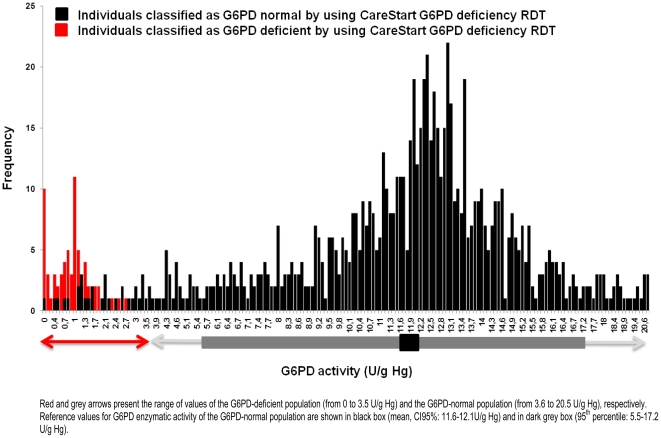
Distribution of the G6PD enzymatic activity (U/g Hb) values, according to the CareStart™ G6PD deficiency screening test results of 903 individuals enrolled in four villages of the Pailin province, Cambodia, 2010.

Distribution of G6PD enzymatic activity of our sampling population according to the results of the CareStart™ G6PD deficiency screening test is given in the Figure 5. Results of the CareStart™ G6PD deficiency screening test and the quantitative G6PD assay are also shown in Table 3.

**Table 3:** Results of the CareStart™ G6PD deficiency screening test® and the quantitative G6PD assay from 903 individuals, Pailin province, Cambodia, 2010.

		Cambodian study data*		Total
		G6PD deficient	G6PD normal	
CareStart G6PD deficiency screening test®	G6PD deficient†	66	0	66
	G6PD normal	31	806	837
Total		97	806	903

*G6PD deficient enzyme activity <3.6 U/g Hb;

† G6PD deficient - no background color change seen in the result window.

The sensitivity of the CareStart™ G6PD deficiency screening test was 0.68 (CI95%: 0.58–0.77) and the specificity was 1.0 (CI95%: 0.99–1.0). Based on the 10.7% G6PD prevalence in our study population, the NLR was 0.32 (CI95%: 0.24–0.43), the PLR could not be calculated, the PPV was 1.0 (CI95%: 0.95–1.0) and the NPV was 0.96 (CI95%: 0.95–0.97). The performance of the CareStart™ G6PD deficiency screening test was not significantly different between genders (data not shown).

A total of 31 false normal results were observed in G6PD deficient individuals with normal Hb concentrations: 4 males with G6PD enzymatic activity was 0–0.9 U/g Hg, eight males and one female with 1–1.9 U/g Hg, nine females and one male with 2–2.9 U/g Hg and four females and three males with 3–3.5 U/g Hg. The threshold of detection for the CareStart™ G6PD deficiency screening test to classifiy people as deficient was below 2.7 U/g Hg.

The evaluation of the long-term temperature stability of the CareStart™ G6PD deficiency screening test showed the test was not affected by high temperatures. Similar performance was found by using tests stored at 35°C or 45°C for 90 days and at 55°C for 72 hours (data not shown).

## Discussion

In many malaria endemic countries like Cambodia that are engaging in malaria elimination, the introduction of primaquine, for transmission blocking or radical cure, in national malaria management guidelines remains a major safety challenge for policy makers. The availability of an accurate, user-friendly, field-adapted RDT for G6PD deficiency would be a major step forward. The main advantages for using this drug are both the prevention of *P. falciparum* transmission from malaria infected patients by killing mature gametocytes and the radical cure *P. vivax* (and *P. ovale*) infections by preventing relapses of the persistent liver parasites [25]. However, it is well-known since 1956 [26], the use of primaquine is not safe in people with erythrocyte glucose-6-phosphate dehydrogenase deficiency. The most common clinical manifestation is an acute hemolytic anaemia. Generally, the residual G6PD enzyme activity correlates with the severity of hemolysis, ranging from mild and transient to severe and life-threatening [15]. Most of these data come from controlled studies in which primaquine was given to G6PD deficient volunteers (especially African A^−^ variants) or normal recipients after transfusion of ^51^Cr-labelled GPD-deficient red cells, and from case reports of patients who developed hemolytic anaemia after receiving primaquine. Therefore, beyond the routine use of primaquine, comprehensive knowledge of the epidemiology of the G6PD deficiency by using reliable methods is needed.

In this study, we have determined, for the first time, the normal reference values of G6PD enzymatic activity for both Cambodian males and females adults by using the quantitative spectrophotometric assay. Reference ranges generally used are derived from North American or European populations which may not be applicable to the Cambodian population. For this purpose, healthy individuals (Khmer ethnic group) were selected with a normal range of hemoglobin concentration and a wild-type sequence in their G6PD gene.

Surprisingly, we found that globally G6PD enzymatic activity was significantly higher in males compared to females (12.5 vs. 11.3 U/g Hg, *P* = 0.005) while previous reports showed no difference using the same methodology [27]. Using these values, the global prevalence of G6PD deficiency in our sample population was 10.7%, ranging from 9.3% in the most southern village to 20% in the most northern village of the Pailin province. The cut-off point to diagnose G6PD deficiency both in Cambodian males and females was around 30% of the normal mean value. As expected [28], we found that the prevalence of G6PD deficiency was almost 2-fold higher in males (15.0%) compared to females (6.9%). This proportion was similar to previous reports from Cambodia [16], [17], [18], [29] and in the bordering countries of Vietnam, Laos and Thailand [28].

Molecular analysis of G6PD variants in deficient people indicated that the most prevalent variant was G6PD-ViangChan, in concordance with previous reports showing that G6PD-ViangChan variant is relatively homogeneous in Cambodians and Laotians in the Eastern part of the Southeast Asian peninsular while G6PD-Mahidol variant is predominant in Myanmar population in the Western part of the peninsular. Moreover, all cases of G6PD-ViangChan (871G>A) were linked to 1311C>T on exon 11 and T>C in nt93 on IVS11, as previously described in neighboring countries [27], [30], [31]. In addition, 1311C>T on exon 11 and T>C in nt93 on IVS11 mutations were not only restricted to G6PD-ViangChan variant since they were observed in 42.8% of people presenting wild type sequence in their G6PD gene, supporting the notion that these sites are highly polymorphic and the occurrence of the mutations is widespread. G6PD-Union (1360C>T) and G6PD-Coimbra (592C>T) which has been already detected in G6PD-deficient Cambodians were not identified [16], [18] but contrary to these two previous reports we observed for the first time in Cambodia G6PD-Mahidol (487G>A), G6PD Canton (1376G>T) and G6PD-Valladolid (406 C>T).

In our study, we have shown that the CareStart™ G6PD deficiency screening test had relatively low sensitivity and high specificity. The kit was able to detect G6PD deficiency at an enzyme activity threshold of <2.7 U/g Hg. This represents a cut-off value of 22% of G6PD normal activity and translates into an ability to detect individuals with severe enzyme deficiency (WHO Classes I and II and some Class III variants). This performance was similar to that classically described for the fluorescent spot method, method recommended since 1979 as screening test, by the International Committee for Standardization in Hematology [32]. Individuals with intermediate or mild G6PD deficiency (from 3.6 to 2.7 U/g Hg, 22%–30% of normal activity) were not identified as deficient by the RDT. For instance, only 40% of G6PD-ViangChan heterozygote females were identified as deficient. Whether this would pose a hazard or an advantage in screening for primaquine therapy depends upon the relative sensitivity to primaquine exhibited by such patients. This important question requires clinical investigation as part of the validation process of this RDT.

The 13 false negative results (12 males and one female) with a G6PD enzymatic activity <2 U/g Hg require careful consideration as an important safety issue. Such individuals would inappropriately receive primaquine and be at risk of clinically significant hemolysis. We systematically classified test results showing any purple color, even if pale, as normal. This may explain the false negatives reported here, and if so, a more conservative approach of classifying ambiguous RDT results as deficient would perhaps have improved sensitivity and diminished specificity. As a clinical safety issue, that would be the preferred approach. In any event, the developers of this RDT may consider a design of that permits visualization of both positive & negative control color changes on each cassette. Assuming the sensitivity reported here may be thus dramatically improved, the CareStart™ G6PD deficiency screening test appears otherwise ideal for use in endemic zones, in particular its apparent robust performance after prolonged storage at high temperatures.

In conclusion, the CareStart™ G6PD deficiency screening test is a new tool under development for screening G6PD deficiency in the field which presents all the advantages of a point-of-care such as fast and easy to perform, without requiring electricity or specific equipment. The first evaluation of this test “outside of the laboratory” showed its capability of detecting severe deficiency as the fluorescent spot test but improvement in sensitivity is recommended. In parallel, more clinical data on the optimal primaquine doses for malaria elimination are needed urgently in order to improve the developpement of the next generation of tests which will aim at providing a reliable and safe “go vs. no-go” decision to safetly use primaquine in routine.
